# Apogossypol-mediated reorganisation of the endoplasmic reticulum antagonises mitochondrial fission and apoptosis

**DOI:** 10.1038/s41419-019-1759-y

**Published:** 2019-07-08

**Authors:** Govindaraju Yedida, Mateus Milani, Gerald M Cohen, Shankar Varadarajan

**Affiliations:** 1 Departments of Molecular and Clinical Cancer Medicine, University of Liverpool, Ashton Street, Liverpool, L69 3GE UK; 2 Molecular and Clinical Pharmacology, University of Liverpool, Ashton Street, Liverpool, L69 3GE UK

**Keywords:** Cell death, Membrane trafficking

## Abstract

The endoplasmic reticulum (ER) with its elaborate network of highly curved tubules and flat sheets interacts with several other organelles, including mitochondria, peroxisomes and endosomes, to play vital roles in their membrane dynamics and functions. Previously, we identified structurally diverse chemicals from different pharmacological classes, which induce a reversible reorganisation of ER membranes. Using apogossypol as a prototypic tool compound, we now show that ER membrane reorganisation occurs at the level of ER tubules but does not involve ER sheets. Reorganisation of ER membranes prevents DRP-1-mediated mitochondrial fission, thereby antagonising the functions of several mitochondrial fission-inducing agents. Previous reports have suggested that ER membranes mark the constriction sites of mitochondria by localising DRP-1, as well as BAX on mitochondrial membranes to facilitate both mitochondrial fission and outer membrane permeabilisation. Following ER membrane reorganisation and subsequent exposure to an apoptotic stimulus (BH3 mimetics), DRP-1 still colocalises with the reorganised ER membranes but BAX translocation and activation, cytochrome *c* release and phosphatidylserine externalisation are all inhibited, thereby diminishing the ability of BH3 mimetics to induce the intrinsic apoptotic pathway. Strikingly, both ER membrane reorganisation and its resulting inhibition of apoptosis could be reversed by inhibitors of dihydroorotate dehydrogenase (DHODH), namely teriflunomide and its active metabolite, leflunomide. However, neither genetic inhibition of DHODH using RNA interference nor metabolic supplementation with orotate or uridine to circumvent the consequences of a loss of DHODH activity rescued the effects of DHODH inhibitors, suggesting that the effects of these inhibitors in preventing ER membrane reorganisation is most likely independent of their ability to antagonise DHODH activity. Our results strengthen the hypothesis that ER is fundamental for key mitochondrial functions, such as fusion-fission dynamics and apoptosis.

Endoplasmic reticulum (ER) with its ribosome-studded sheets and reticulated tubules has long been known as the principal site of protein synthesis, intracellular calcium storage and lipid synthesis^1–5^. Alterations in these functions triggers a stress response, commonly referred to as the unfolded protein response (UPR)^6^. Over the last decade, the physical interactions of the ER network with numerous subcellular organelles and the physiological consequences of such interactions have been extensively studied. To date, the ER has been implicated in several diverse functions, including the regulation of mitochondrial membrane dynamics^7,8^, endosome fission and positioning^9,10^, autophagosome biogenesis^11^, peroxisomal motility^12,13^, store-operated calcium entry^14^ and regulation of reactive oxygen species^15^.

Previously, we have reported an evolutionarily conserved, novel form of ER stress response, characterised by a reversible reorganisation of ER membranes, following exposure of cells to a wide variety of drugs from distinct chemical classes^16^. Using one such drug, apogossypol, as a tool compound, we characterised such ER membrane reorganisation to be distinct from canonical ER stress and the classical unfolded protein response (UPR). However, such reorganisation of ER membranes resulted in defects in ER-golgi trafficking and global protein synthesis^16^. Finally, we also observed that changes in ionic fluxes, and in particular, influx of sodium ions could regulate ER membrane reorganisation^17^.

Recently, it has been reported that the physical interaction between ER and mitochondria plays a key role in mitochondrial mediated functions such as fission/fusion dynamics, DRP-1 recruitment and potentially apoptosis^7,8^. In this report, we show that the reversible reorganisation of ER tubules prevents mitochondrial fission, recruitment and activation of BAX, outer mitochondrial membrane permeabilisation as well as apoptosis.

## Materials and methods

### Cell culture

HeLa cells (purchased from ATCC, Middlesex, UK) were grown in DMEM medium (Life Technologies Inc., Paisley, UK). H1299 (purchased from ATCC), KCL22 (provided by Prof. R. Clark, University of Liverpool) and MAVER-1 cells (provided by Dr. J. Slupsky, University of Liverpool) were cultured in RPMI 1640 medium (Life Technologies). H929 cells (purchased from DMSZ, Braunshweig, Germany) were cultured in RPMI 1640 medium supplemented with 0.05 mM β-mercaptoethanol (BME). All culture media were supplemented with 10% FBS (Life Technologies) and maintained at 37 °C in a humidified atmosphere of 5% CO_2_. All cell lines used in this study were subjected to short tandem repeat (STR) profiling to ensure quality and integrity.

### Reagents

Apogossypol and Leflunomide from ApexBio (Boston, MA, USA), A-1331852, A-1210477, ABT-199, Z-VAD.FMK and CCCP from Selleck (Houston, TX, USA), Teriflunomide, norhydroguaiaretic acid (NDGA), Ivermectin, Terfenadine, Suloctidil, orotate and uridine from Sigma Aldrich (St Louis, MO, USA), MitoTracker Deep Red FM from Thermo Fisher (Loughborough, UK) were used. Antibodies against BAP31, RTN4, BiP, PDI, CHOP, DHODH and tubulin from Abcam (Cambridge, UK), CLIMP-63 and BAX (6A7) from Enzo Life Sciences (Exeter, UK), TIM22 and KNT-1 from Sigma, HSP60, Cytochrome *c*, BAX, OPA1 and DRP-1 from BD Biosciences (San Jose, CA, USA), phospho-DRP-1 (S616), phospho-DRP-1 (S637), MFN1 and MFN2 from Cell Signaling Technologies (Danvers, MA, USA), BAK (AB-1) from Calbiochem (Watford, UK), MFF, MID49 and MID51 from ProteinTech (Manchester, UK) and GAPDH from Santa Cruz Biotechnologies (Santa Cruz, CA, USA) were used. For RNA interference, cells were transfected with 10 nM of siRNAs against DHODH (SI00363384 and SI00363391) purchased from Qiagen Ltd. (Manchester, UK), using Interferin (Polyplus Transfection Inc, NY), according to the manufacturer’s protocol and processed 72 h after transfection.

### Microscopy

For electron microscopy, cells were fixed and processed as previously described. Electron micrographs were recorded using an ES1000W CCD camera and Digital Micrograph software (Gatan, Abingdon, UK) with a Zeiss 902A electron microscope or with a Megaview 3 digital camera and iTEM software (Olympus Soft Imaging Solutions GmbH, Münster, Germany) in a Jeol 100-CXII electron microscope (Jeol UK Ltd., Welwyn Garden City, UK). For immunocytochemistry, cells grown on coverslips were fixed with 4% (w/v) paraformaldehyde, permeabilised with 0.5% (v/v) Triton X-100 in PBS, followed by incubations with primary antibodies, the appropriate fluorophore-conjugated secondary antibodies, mounted on glass slides and imaged using a 3i Marianas spinning disk confocal microscope, fitted with a Plan-Apochromat ×63/1.4NA Oil Objective, M27 and a Hamamatsu ORCA-Flash4.0 v2 sCMOS Camera (all from Intelligent Imaging Innovations, GmbH, Germany).

### Cytochrome *c* release assay

In total 3 × 10^6^ cells were washed in cold PBS and resuspended in mitochondrial isolation buffer (250 mM sucrose, 20 mM HEPES, pH 7.4, 5 mM MgCl_2_ and 10 mM KCl) containing 0.05% digitonin. Cells were left on ice for 10 min followed by centrifugation at 13,000 × *g* for 3 min. Subsequently, supernatant and pellets were analysed by western blotting.

### Flow cytometry

Apoptosis was assessed by measuring the extent of phosphatidylserine (PS) externalisation in cells exposed to the relevant drugs, following staining with Annexin V-FITC, in annexin binding buffer (150 mM NaCl, 10 mM HEPES pH 7.4, 1 mM MgCl_2_, 5 mM KCl, 1.8 mM CaCl_2_) and propidium iodide (5 µg/ml) and subjected to flow cytometry. To measure BAX and BAK activation, cells were exposed to the indicated treatments, collected and fixed with 2% paraformaldehyde at room temperature for 10 min. Fixed cells were then washed with PBS and re-suspended in permeabilisation buffer (0.1% saponin, 0.5% BSA) for 10 min, followed by incubation with the corresponding primary antibodies (BAX 6A7 or BAK AB-1) and fluorophore-labelled secondary antibodies. Activated BAX or BAK was then detected using flow cytometry.

### Western blotting

Western blotting was carried out according to standard protocols. Briefly, 50 μg of total protein lysate was subjected to SDS-PAGE electrophoresis. Subsequently proteins were transferred to nitrocellulose membrane and protein bands visualised with ECL reagents (GE Healthcare).

### Statistical Analysis

One-way ANOVA with Bonferroni’s multiple comparison test was performed to evaluate differences between conditions. Asterisks depicted correspond to the following *p* values: *p ≤ 0.05, **p ≤ 0.005 and ***p ≤ 0.001.

## Results

### Apogossypol-induced ER membrane reorganisation involves ER tubules and not sheets

**Fig. 1:**
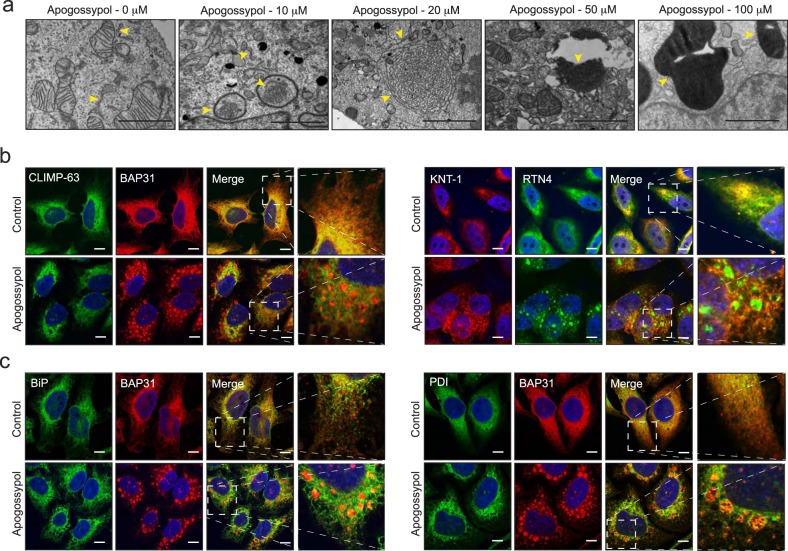
Apogossypol induces a concentration-dependent reorganisation of ER tubules and not sheets. **a** HeLa cells exposed to apogossypol for 4 h exhibited a marked concentration-dependent reorganisation of ER membranes (yellow arrowheads). Scale bar: 2 μm. **b** HeLa cells were exposed to apogossypol (20 μΜ) for 4 h and immunostained with the indicated antibodies. The boxed regions in the images are enlarged on the right to show the extent of colocalisation. Scale bar: 10 μm

Previously, we reported a non-canonical form of ER stress induced by several drugs from distinct chemical classes^16^. Using one of those drugs, apogossypol, as a tool compound, we now further characterise the nature of this novel form of ER stress. In HeLa cells, exposure to apogossypol resulted in extensive reorganisation of ER membranes (denoted by the yellow arrowheads) that occurred in a concentration-dependent manner (Fig. 1a). At high concentrations (>50 μM) of apogossypol, the ER membranes were densely packed and the reticular ER membranes were no longer detectable (Fig. 1a). Immunolabeling of ER tubule markers, BAP31 and RTN4 revealed extensive redistribution of these proteins on the reorganised ER membranes (Fig. 1b). In marked contrast, markers that exclusively detect ER sheets (CLIMP-63 and KTN-1)^2^ or ER lumen (BiP and PDI) failed to colocalise with the reorganised ER membranes (Fig. 1b, c), suggesting that exposure of the cells to apogossypol results in the exclusive reorganisation of ER tubules and not sheets.

### Apogossypol-mediated ER membrane reorganisation antagonises mitochondrial fission mediated by distinct stimuli

**Fig. 2:**
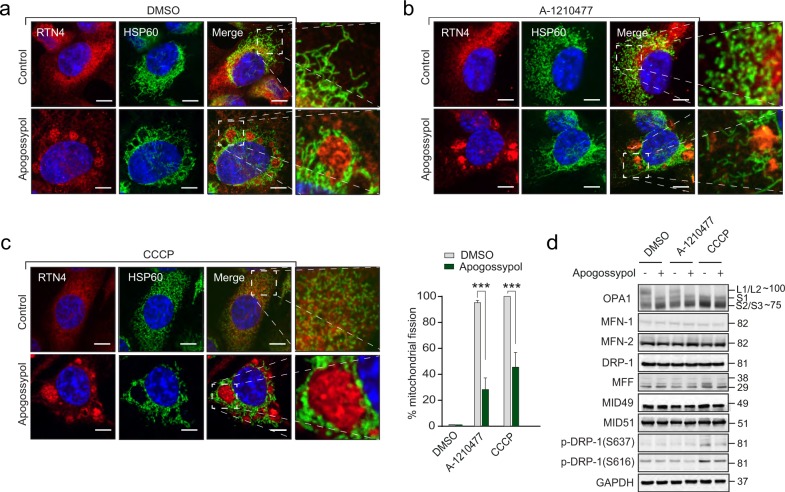
Apogossypol-mediated ER membrane reorganisation antagonises mitochondrial fission. H1299 cells were exposed to apogossypol (20 μΜ) for 1 h, followed by either **a** DMSO, **b** A-1210477 (10 µM) for 4 h or **c** CCCP (20 μΜ) for 1 h and assessed for mitochondrial membrane integrity and ER membrane reorganisation by immunostaining with HSP60 and RTN4 antibodies, respectively. Scale bar: 10 μm. The boxed regions in the images are enlarged on the right to show mitochondrial structural changes in the indicated cells. Quantification of mitochondrial fission was performed by counting ~100 cells from 3 independent experiments. Error bars = Mean ± SEM. ***p ≤ 0.001. **d** Western blot analysis of whole cell lysates of H1299 cells exposed to apogossypol (20 μΜ) in the presence and absence of A-1210477 or CCCP

Recent studies have highlighted the importance of ER membranes in marking the initial site of mitochondrial membrane fission^7,8^. These observations together with our report of an MCL-1 inhibitor (A-1210477), inducing extensive, DRP-1-mediated mitochondrial fission in several cell lines, including H1299, a non-small cell lung carcinoma cell line^18^, led us to question whether ER membrane reorganisation would alter A-1210477-mediated mitochondrial fission. Exposure of H1299 cells to apogossypol resulted in extensive reorganisation of the ER membranes with minimal changes to the filamentous network of mitochondrial membranes, as assessed by immunostaining with a mitochondrial marker (HSP70) (Fig. 2a). Exposure to A-1210477 resulted in significant mitochondrial fission, which was markedly inhibited in cells exhibiting extensive ER membrane reorganisation (Fig. 2b). This was not just restricted to A-1210477, as cells with reorganised ER membranes also inhibited CCCP (a mitochondrial proton uncoupler)-mediated mitochondrial fission in these cells (Fig. 2c).

In order to assess whether apogossypol regulated mitochondrial fusion or fission events, expression levels of the different mitochondrial fission-fusion proteins were assessed. Mitochondrial fusion is mediated by GTPases, such as OPA1 and mitofusins-1/2 (MFN1/MFN2)^19^. A loss of fusion results in the proteolytic processing of the long isoforms (L1 and L2) of OPA1 to yield three short isoforms (S1-S3)^20–22^. While A-1210477 did not result in any alterations in OPA1 processing compared to the control cells, exposure to CCCP resulted in OPA1 proteolysis (Fig. 2d). Surprisingly, exposure to apogossypol also resulted in a similar proteolysis of OPA1, despite maintaining normal mitochondrial morphology (Fig. 2a, d). In contrast, no changes in the expression levels of MFN1 and MFN2 or mitochondrial fission GTPase, DRP-1 or its receptors, MFF, MID49 and MID51^23,24^ were evident in these cells (Fig. 2d). Nevertheless, exposure to apogossypol resulted in a small decrease in the phosphorylation status of DRP-1 (at S616, which is associated with mitochondrial fission) in cells exposed to either A-1210477 or CCCP, implicating a role for apogossypol in regulating DRP-1 phosphorylation (at S616) following specific stimuli (Fig. 2d). A similar decrease in phospho-DRP-1 (at S637, which is normally associated with mitochondrial fusion) was observed in cells following a combination of apogossypol and CCCP (Fig. 2d), further complicating the role of apogossypol in DRP-1 phosphorylation and mitochondrial membrane dynamics.

**Fig. 3:**
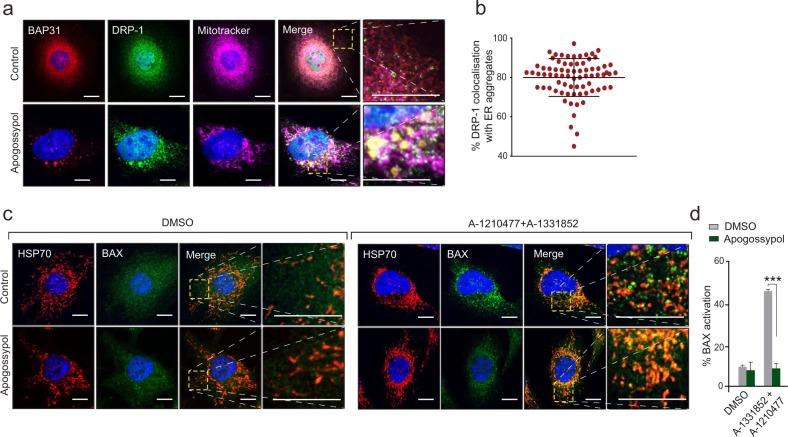
Reorganised ER membranes co-localise with DRP-1 and prevent activation of BAX. **a** HeLa cells were exposed to apogossypol (20 μΜ) for 4 h, stained with Mitotracker and immunostained with DRP-1 and BAP31 antibodies. **b** The extent of DRP-1 that was colocalised to the BAP31-positive reorganised ER tubules was assessed in 80 cells and plotted. Each dot in the graph corresponds to one cell. Error bars = Mean ± SD. **c** HeLa cells were exposed to Z-VAD.fmk (30 μΜ) for 30 min and apogossypol (20 μΜ) for 1 h, followed by a combination of BH3 mimetics, A-1331852 (0.1 μΜ) and A-1210477 (10 μΜ) for 4 h, and immunostained with BAX and HSP70 antibodies. Scale bar: 10 μm. In both **a**, **c**, the boxed regions in the images are enlarged to show the extent of colocalisation. **d** HeLa cells treated as in **c** were stained with BAX (6A7) antibody and the extent of BAX activation assessed by flow cytometry. Graphs were plotted using data from three independent experiments. Error bars = Mean ± SEM. ***p ≤ 0.001

Since mitochondrial translocation of DRP-1 is a prerequisite for mitochondrial fission, we wondered whether the reorganised ER membranes would prevent this translocation. Interestingly, the normal punctate distribution of DRP-1 (that suggests its dynamic shuttling between mitochondria and cytosol) was grossly altered following apogossypol, and most, if not all, DRP-1 appeared to be associated with the reorganised ER membranes (Fig. 3a, b).

### ER membrane reorganisation prevents BAX translocation and activation following BH3 mimetics

DRP-1-mediated mitochondrial fission could be linked to apoptosis induction due to recruitment of BAX to mitochondrial constriction sites by the ER membranes^25,26^. During apoptosis, cytosolic BAX translocates to the mitochondria, where it is activated to form oligomeric channels, that result in mitochondrial outer membrane permeabilisation (MOMP) and the release of cytochrome *c*^27,28^. In agreement, BAX appeared largely cytosolic both under control conditions and following apogossypol-induced ER membrane reorganisation (Fig. 3c), whereas following exposure to a combination of BH3 mimetics (A-1331852; BCL-X_L_ inhibitor and A-1210477)^29,30^, BAX translocated to the mitochondrial membranes, which was characterised by a distinct punctate staining (Fig. 3c). Such punctate distribution of BAX, however, was markedly reduced in cells treated with a combination of apogossypol and BH3 mimetics (Fig. 3c). Consistent with this observation, exposure of cells to apogossypol prevented BH3 mimetic-mediated activation of BAX but not BAK (Fig. 3d and Supplementary Fig. 1), thus confirming that reorganisation of ER membranes prevented BAX translocation and activation.

### ER membrane reorganisation inhibits BH3-mimetic-mediated mitochondrial cytochrome *c* release and apoptosis

**Fig. 4:**
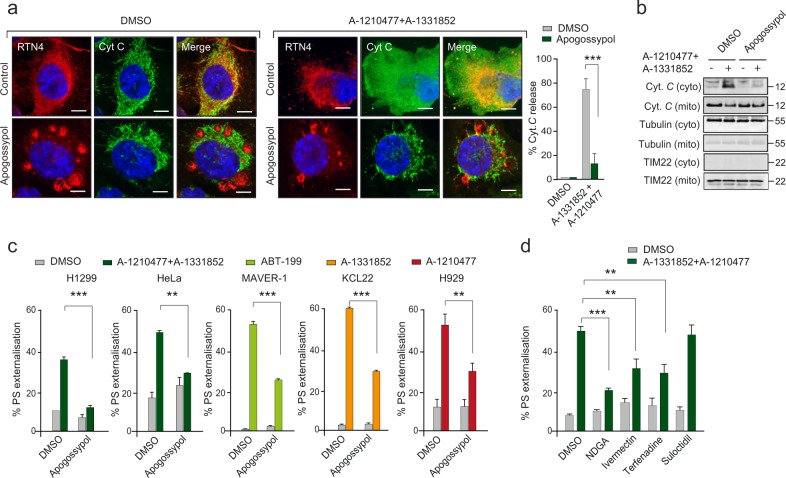
ER membrane reorganisation inhibits BH3 mimetic-mediated mitochondrial outer membrane permeabilisation and apoptosis. **a** H1299 cells exposed to apogossypol (20 μΜ) for 1 h and then exposed to a combination of BH3 mimetics, A-1331852 (0.1 μΜ) and A-1210477 (10 μΜ) for a further 4 h and assessed for cytochrome *c* release and ER morphology by immunocytochemistry. Scale bar: 10 μm. Quantification of the extent of cytochrome *c* release was performed by counting ~100 cells from 3 independent experiments. Error bars = Mean ± SEM. ***p ≤ 0.001. **b** Western blot analysis of cytosolic and mitochondrial fraction of H1299 cells exposed to BH3-mimetics and apogossypol. Tubulin and TIM22 were used as controls for assessing the purity of the cytosolic and mitochondrial fractions. **c** The indicated cell lines were exposed to apogossypol (20 µM) for 1 h, followed by the indicated specific BH3 mimetics for 4 h and then assessed for apoptosis by PS externalisation. **d** HeLa cells exposed to either NDGA (50 µM), Ivermectin (20 µM), Terfenadine (5 µM), or Suloctidil (5 µM) for 1 h, followed by a combination of A-1210477 (10 µM) and A-1331852 (0.1 μΜ) for 4 h were assessed for apoptosis. Error bars = Mean ± SEM. ***p ≤ 0.001 and **p ≤ 0.005

Since apogossypol-induced ER membrane reorganisation prevented BAX activation and mitochondrial translocation, we wished to assess whether it would also affect BH3 mimetic-mediated release of cytochrome *c* and apoptosis. The combination of A-1210477 and A-1331852 resulted in extensive release of cytochrome *c* from the mitochondria to the cytosol, as detected both by immunocytochemistry and western blot analyses, which was markedly inhibited by pretreatment of cells with apogossypol (Fig. 4a, b). Furthermore, exposure to apogossypol significantly diminished BH3 mimetic-mediated apoptosis in cells that depend for survival either on both BCL-X_L_ and MCL-1 (H1299 and HeLa), or exclusively on BCL-2 (MAVER-1), BCL-X_L_ (KCL22) and MCL-1 (H929)^31–33^ (Fig. 4c). Finally, to investigate whether it was apogossypol-mediated ER membrane reorganisation or an unrelated effect of apogossypol that was responsible for the anti-apoptotic effect, HeLa cells were exposed to structurally diverse ER membrane reorganising drugs, such as NDGA, ivermectin, terfenadine and suloctidil^16^. While the first three drugs resulted in both an extensive reorganisation of ER membranes and protection (to varying degrees) against BH3 mimetic-mediated apoptosis, suloctidil failed to protect against BH3 mimetic-mediated apoptosis (Fig. 4d). The protective effects of the different agents mimicked their abilities to induce ER membrane reorganisation in cells (Supplementary Fig. 2), thus probably explaining why suloctidil was not as potent as the other agents in protecting against BH3 mimetic-mediated apoptosis. Taken together, our data convincingly demonstrated that ER membrane reorganisation antagonised BH3 mimetic-mediated apoptosis and changes in mitochondrial structure.

### Teriflunomide and Leflunomide prevent apogossypol-mediated ER membrane reorganisation

**Fig. 5:**
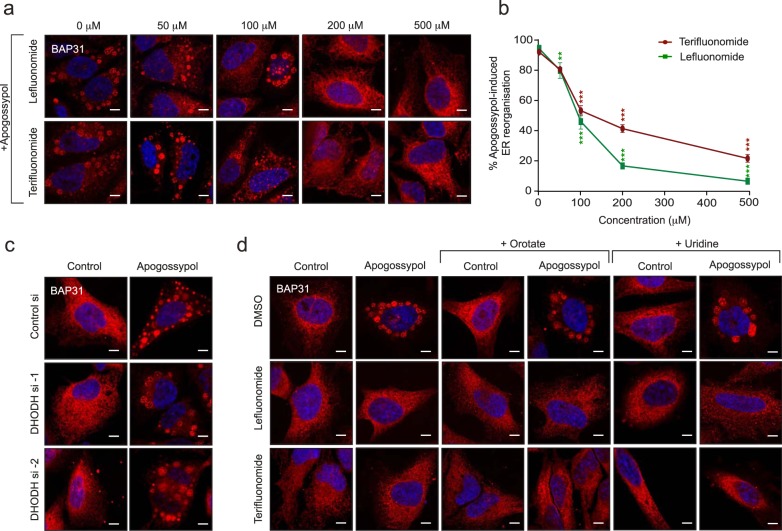
Teriflunomide and leflunomide prevent ER membrane reorganisation independent of their ability to inhibit DHODH. **a** HeLa cells were exposed to increasing concentrations of teriflunomide or leflunomide for 1 h, followed by apogossypol (20 μΜ) for 4 h and immunostained with BAP31 antibody. **b** Quantification of the reduction in ER membrane reorganisation was performed by counting ~300 cells from 3 independent experiments. Error bars = Mean ± SEM. ***p ≤ 0.001. **c** HeLa cells transiently transfected with two different siRNAs against DHODH for 72 h were exposed to apogossypol (20 μΜ) for 4 h, and then immunostained with BAP31 antibody. **d** HeLa cells were supplemented with excess orotate or uridine (1 mM each), followed by inhibition of DHODH (teriflunomide or leflunomide at 200 μΜ) for 1 h, and a further 4 h with apogossypol (20 μΜ), before immunostaining with BAP31 antibody. Scale bar: 10 μm

Previously, we observed that an inhibitor of store-operated calcium entry (SOCE), 2-APB (2-aminoethoxydiphenylborate) was extremely effective in preventing apogossypol-mediated ER reorganisation^17^. Other modulators of SOCE, such as teriflunomide and leflunomide^34^, in addition to inducing canonical ER stress and the UPR^35^ (Supplementary Fig. 3), resulted in a concentration-dependent decrease of apogossypol-mediated ER membrane reorganisation (Fig. 5a, b), with leflunomide being slightly more potent than teriflunomide. However, silencing the expression levels of DHODH using two different siRNAs did not affect apogossypol-mediated ER membrane reorganisation (Fig. 5c and Supplementary Fig. 4), suggesting that the inhibitors most likely prevented apogossypol-mediated ER membrane reorganisation independent of their ability to inhibit DHODH. Therefore, we performed metabolic supplementation studies with orotate or uridine to circumvent the need for cells to rely on DHODH for pyrimidine synthesis. Under control conditions, in the absence of the supplements, exposure of cells to teriflunomide and leflunomide abolished apogossypol-mediated ER membrane reorganisation and this was unaffected by supplementation with orotate or uridine (Fig. 5d). These results strongly suggest that teriflunomide and leflunomide most likely function in a DHODH-independent manner to prevent ER membrane reorganisation.

### Teriflunomide and Leflunomide prevent the anti-apoptotic function of apogossypol-mediated ER membrane reorganisation

**Fig. 6:**
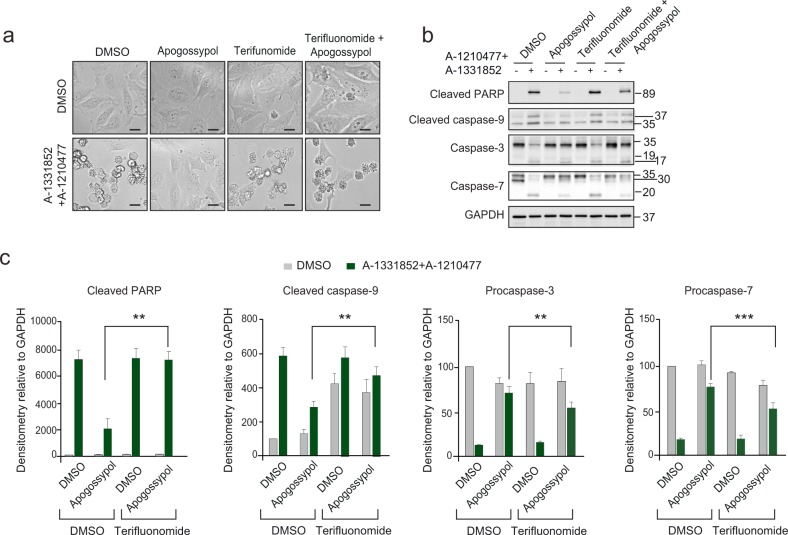
Teriflunomide reverses the anti-apoptotic effect of apogossypol and enhances BH3 mimetic-mediated apoptosis. **a** HeLa cells were exposed to teriflunomide (200 μΜ) for 1 h, followed by apogossypol (20 μΜ) for another 1 h, and finally to a combination of BH3 mimetics, A-1331852 (0.1 μΜ) and A-1210477 (10 μΜ) for 4 h and assessed for cell death. **b** Western blots from cells treated in **a** indicate changes in the extent of processing of caspase-9, −3 and −7 as well the cleavage of the caspase substrate, PARP. **c** Densitometric analyses of the different proteins from the blots shown in **b** were performed using ImageJ software. The values for DMSO controls were normalised to 100%, and the densitometry of the rest plotted relative to the intensities of GAPDH (protein loading control) in the corresponding lanes. Graphs were plotted using data from three independent experiments. Error bars = Mean ± SEM. ***p *≤* 0.001 and **p *≤* 0.005

**Fig. 7:**
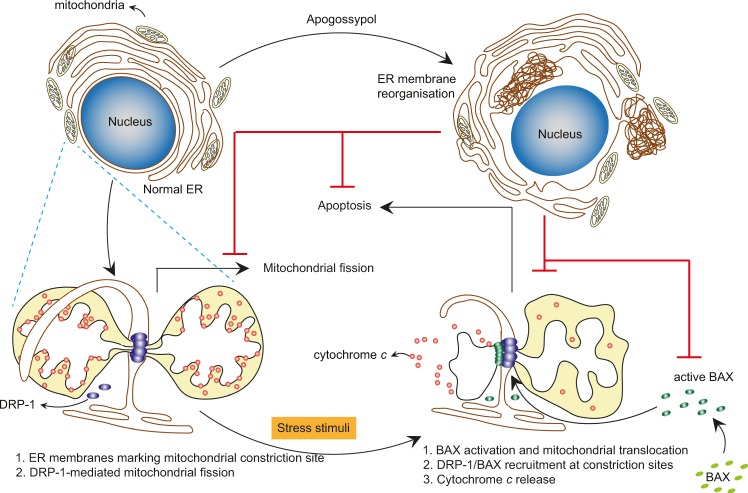
Apogossypol-mediated ER membrane reorganisation antagonises mitochondrial fission, MOMP and apoptosis. Apogossypol induces ER membrane reorganisation (top part of the figure). ER membranes are normally in close proximity to mitochondria and mark mitochondrial constriction sites. This is illustrated in the left lower part of the figure, which depicts ER membranes wrapping around a mitochondrion at the constriction site. This site is also marked by the recruitment of DRP-1 monomers, which ultimately form an oligomeric constriction ring thereby facilitating mitochondrial fission. Upon exposure to an apoptotic stress stimulus, cytosolic BAX is activated and is also recruited onto the constriction sites, facilitating MOMP and cytochrome *c* release, resulting in the induction of apoptosis. Apogossypol-induced ER membrane reorganisation antagonises mitochondrial fission, BAX activation, recruitment on mitochondrial membranes, caspase activation and apoptosis

While exposure of cells to apogossypol resulted in diminished BH3 mimetic-mediated apoptosis, this was reversed when cells were pre-treated with teriflunomide (Fig. 6a). This rescue of cell death from apoptosis was observed also in the levels of caspase activation and activity (Fig. 6b). In cells exposed to BH3 mimetics alone, the appearance of processed caspase-9 and cleaved PARP was accompanied by the extensive processing of intact caspase-3 and -7 to their active processed forms (Fig. 6b). PARP cleavage and the accompanying loss of procaspase-3 following BH3 mimetics were largely prevented upon exposure to apogossypol (Fig. 6b). This rescue from apoptosis was reversed when cells were also treated with teriflunomide, as demonstrated by the densitometry plots of the western blots (Fig. 6c). Collectively, this study demonstrates that apogossypol-mediated reorganisation of ER tubules prevents the recruitment of DRP-1 and BAX to mitochondrial membranes during apoptosis, which in turn results in defects in mitochondrial fission, outer mitochondrial membrane permeabilisation and apoptosis (Fig. 7).

## Discussion

Although several fission and fusion GTPases that regulate membrane dynamics of mitochondria have been characterised^36^, the role of the ER in marking the sites of mitochondrial fission has only recently been characterised^7,8,37^. Our previous observations of a noncanonical form of ER stress, characterised by the reversible reorganisation of ER membranes^16^, led us to ask what effects such ER reorganisation would have on mitochondrial structure and function. We describe for the first time that ER membrane reorganisation was restricted to ER tubules and not ER sheets (Fig. 1). In this study, we were unable to assess whether ER membrane reorganisation altered fission-fusion dynamics of mitochondria in control cells, as the mitochondria in these cells were largely filamentous. However, apogossypol antagonised mitochondrial fission mediated by specific fission inducers (Fig. 2), which is likely due to the redistribution of DRP-1 to the reorganised ER membranes (Fig. 3).

In addition, apogossypol-mediated ER membrane reorganisation also prevented BH3 mimetic-mediated apoptosis (Fig. 4), which was surprising as apogossypol was originally developed as a pan-BCL-2 inhibitor to induce apoptosis in cancer cells^38,39^. However later studies showed that apogossypol, unlike more specific BH3 mimetics, induced a BAX/BAK-independent cell death following prolonged exposure (>24 h)^40,41^. In marked contrast, apogossypol-mediated ER reorganisation is an early event occurring within 30 min of exposure^16^. In this study, apogossypol was merely used as a tool compound to induce ER membrane reorganisation. Furthermore, other ER membrane reorganising agents also protected against BH3 mimetic-mediated apoptosis (Fig. 4), thereby implicating ER membrane reorganisation as a modulator of mitochondrial fission and apoptosis. Although the involvement of DRP-1 in regulating apoptosis have previously been reported^8,18,25,42^, to our knowledge, this is one of the first reports discussing the role of ER membranes in BH3 mimetic-mediated apoptosis.

Apogossypol prevented BH3 mimetic-mediated apoptosis by preventing the activation of BAX but not BAK (Fig. 3 and Supplementary Fig. 1). This also indicated that activated BAK alone was insufficient to induce apoptosis in these cells. In support of this suggestion, HCT-116 cells, which express both BAX and BAK, undergo BH3 mimetic-induced apoptosis in a BAX- but not BAK-dependent manner^31,43^. Similarly, BAK is required for BH3 mimetic-induced apoptosis, only when BAX is not available, as evident in H1299 and Jurkat-T-lymphocytes^18,41^. Since HeLa cells express both BAX and BAK, it is possible that these cells rely on BAX more than BAK to induce BH3 mimetic-mediated apoptosis.

ER membrane reorganisation has recently been reported in dengue virus-infected cells, in which the virus antagonises mitochondrial fission by dephosphorylating DRP-1 at Ser-616 to dampen the innate immune response, thus favouring viral replication^44^. This could well be true in other viral infections and antagonising ER reorganisation in such instances could facilitate mitochondrial fission and enhance cell death of the virus-infected cells. Measures to identify agents that can reverse ER membrane reorganisation resulted in the observation that 2-APB, an inhibitor of store-operated calcium entry (SOCE) was effective in antagonising apogossypol-mediated ER membrane reorganisation^17^. This is in agreement with previous reports that have implicated a critical role for Ca^2+^ transfer between ER and mitochondria to regulate ER-mitochondria contacts, as well mitochondrial fission^45–48^. Since pre-treatment with 2-APB prevented apogossypol-mediated ER reorganisation, we wished to assess whether the corresponding anti-apoptotic effects against BH3 mimetics could also be reversed. However, 2-APB was cytotoxic at concentrations (~100 μM) required to diminish apogossypol-mediated ER reorganisation (data not shown) and hence could not be used in this context. However, a recent report that screened a large panel of FDA-approved drugs to identify modulators of SOCE revealed that inhibitors of DHODH, teriflunomide and leflunomide, were effective at inhibiting SOCE at clinically relevant doses^34^. As expected, these inhibitors were potent in antagonising ER membrane-mediated reorganisation and its protective effects against BH3 mimetic-mediated apoptosis, however such protective effects appeared to be independent of their ability to inhibit DHODH. Although the precise mechanisms whereby DHODH inhibitors antagonise apogossypol-mediated ER membrane reorganisation are unclear, these inhibitors can be used as tool compounds to uncouple the anti-apoptotic effects of ER membrane reorganisation and potential off-target effects of apogossypol in antagonising apoptosis. Furthermore, in a therapeutic context, these inhibitors may have the potential to dampen replication of dengue virus and enhance the innate immune response of the host.

## Supplementary information

Figure S1

Figure S2

Figure S3

Figure S4

Supplementary Legends
