# Supplementary figures and images for: Apogossypol-mediated reorganisation of the endoplasmic reticulum antagonises mitochondrial fission and apoptosis
Source: Cell Death Dis. 2019 Jul 8;10(7):521. doi: 10.1038/s41419-019-1759-y (PMC6614446; doi:10.1038/s41419-019-1759-y)

Figure S1

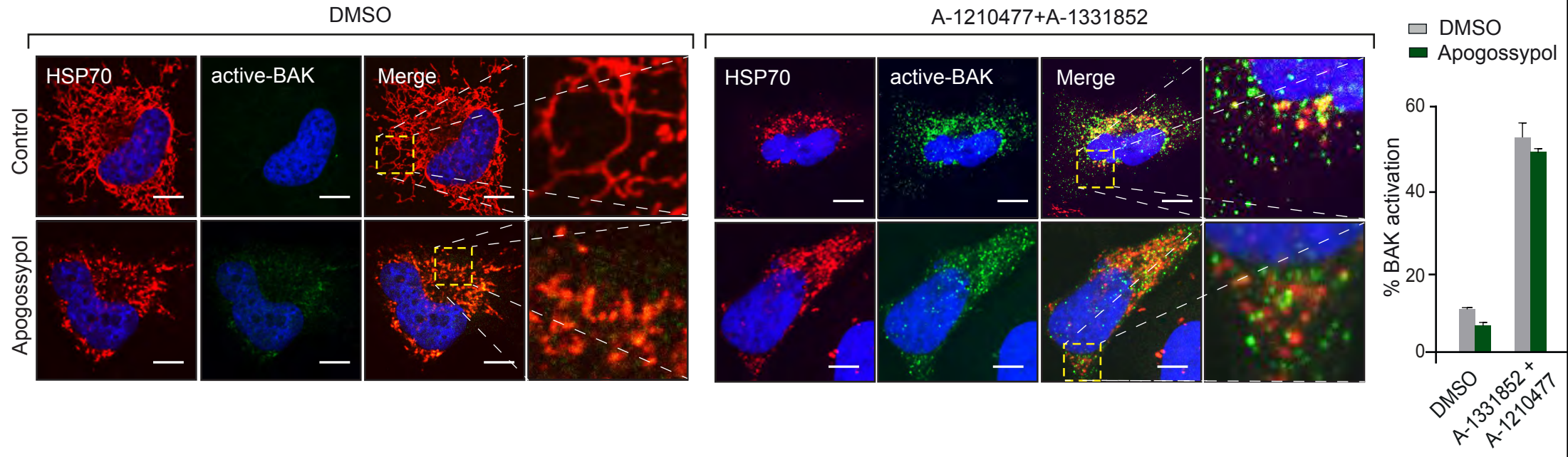

Supplement: Supplementary file 1 — Figure S1 [file 41419_2019_1759_MOESM1_ESM.pdf]

Figure S2

a

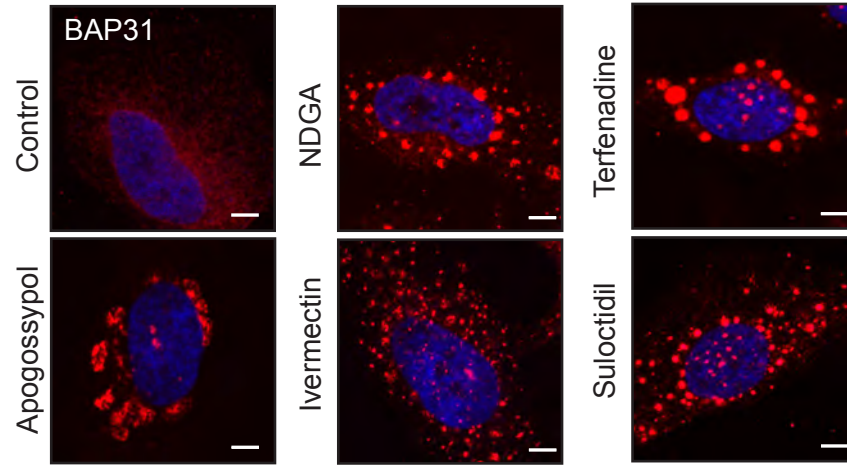

b

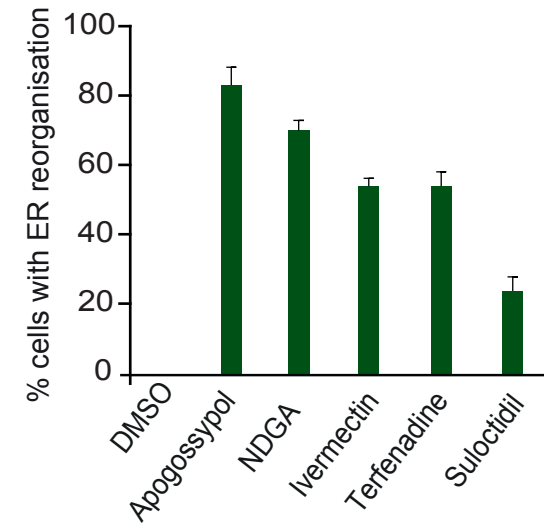

Supplement: Supplementary file 2 — Figure S2 [file 41419_2019_1759_MOESM2_ESM.pdf]

### Figure S3

a

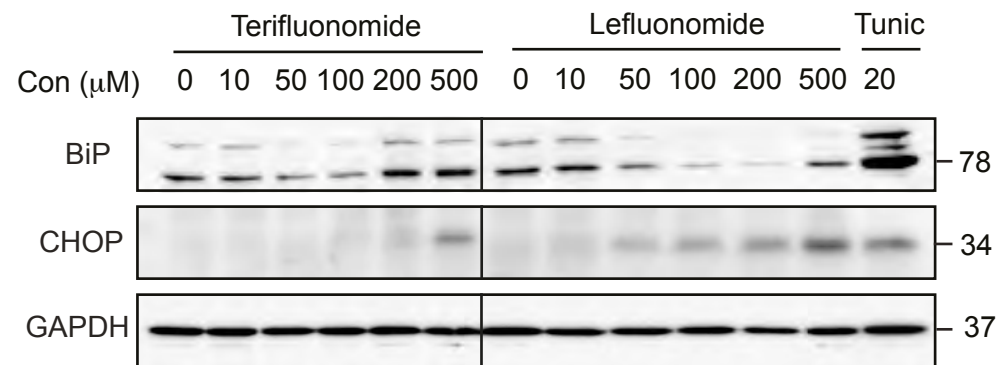

**b**

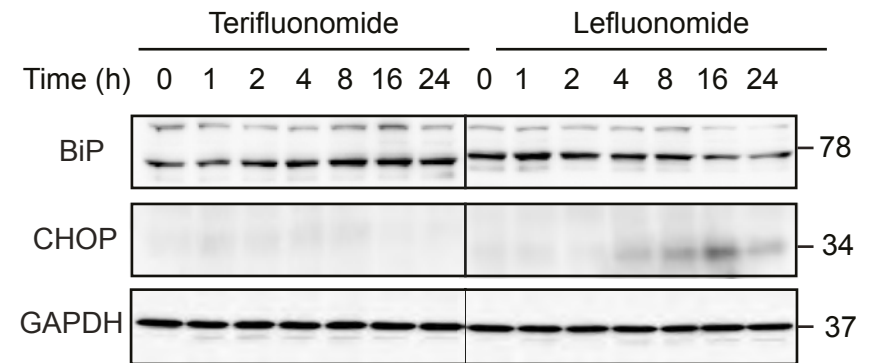

Supplement: Supplementary file 3 — Figure S3 [file 41419_2019_1759_MOESM3_ESM.pdf]

# Figure S4

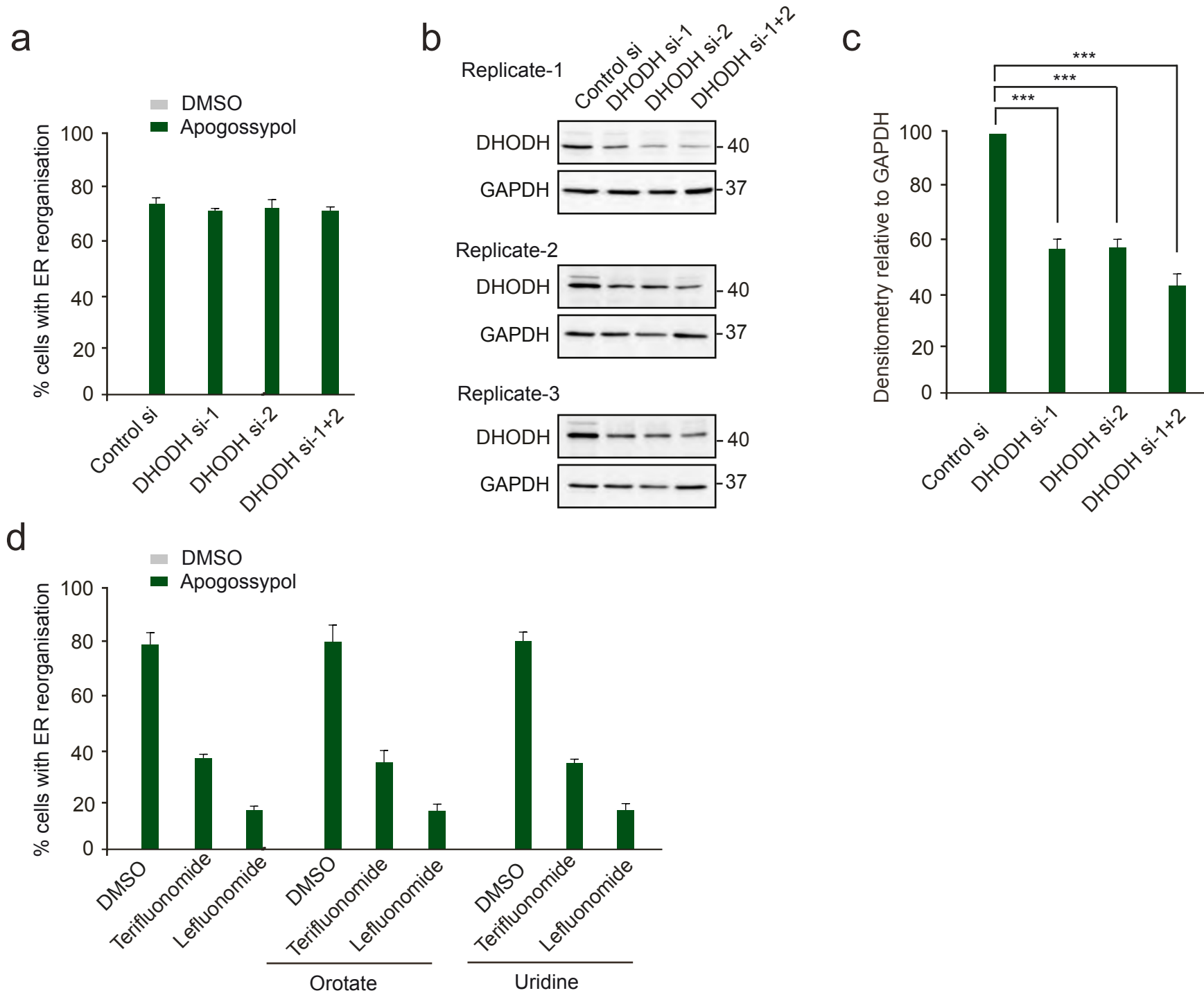

Supplement: Supplementary file 4 — Figure S4 [file 41419_2019_1759_MOESM4_ESM.pdf]
