# Supplementary material for: Apogossypol-mediated reorganisation of the endoplasmic reticulum antagonises mitochondrial fission and apoptosis
Source: Cell Death Dis. 2019 Jul 8;10(7):521. doi: 10.1038/s41419-019-1759-y (PMC6614446; doi:10.1038/s41419-019-1759-y)
Supplement: Supplementary file 5 — Supplementary Legends [file 41419_2019_1759_MOESM5_ESM.docx]

**Supplementary Information**

**Fig. S1. Apogossypol does not alter BH3 mimetic-mediated activation of BAK.** (a) HeLa cells were exposed to Z-VAD.fmk (30 μM) for 30 minutes and apogossypol (20 μM) for 1 h, followed by a combination of BH3 mimetics, A-1331852 (0.1 μM) and A-1210477 (10 μM) for 4 h, and immunostained with active BAK and HSP70 antibodies. Scale bar: 10 μm. The boxed regions in the images are enlarged to show the extent of BAK activation. (b) HeLa cells treated as in (a) were stained with active BAK (AB-1) antibody and activation assessed by flow cytometry. Graphs were plotted using data from three independent experiments. Error bars=Mean ± SEM.

**Fig. S2. Drugs from distinct chemical classes induce ER membrane reorganisation.** HeLa cells were exposed to either apogossypol (20 μM), NDGA (50 µM), Ivermectin (20 µM), Terfenadine (5 µM) or Suloctidil (5 µM) for 4 h and immunostained with BAP31 antibody. Scale bar: 10 μm.

**Fig. S3. DHODH inhibitors induce the unfolded protein response in a concentration- and time-dependent manner.** Whole cell lysates of HeLa cells exposed to either (a) increasing concentrations for 24 h or (b) a fixed concentration (200 μM) of the DHODH inhibitors for the indicated times, were probed for changes in expression levels of BiP and CHOP, to measure changes in the unfolded protein response. GAPDH was used as a loading control and cells exposed to Tunicamycin (20 μM) for 24 h was used as a positive control.

**Fig. S4. Genetic knockdown of DHODH using RNA interference was not very effective.** (a) HeLa cells, transiently transfected with two different siRNAs against DHODH for 72 h and exposed to apogossypol (20 μM) for 4 h, were immunostained with BAP31 antibody and the extent of ER membrane reorganisation quantified in at least 300 cells from 3 independent experiments. Error bars = Mean ± SEM. (b) Western blots of cells from (a) show the knockdown efficiency of the two different DHODH siRNAs in 3 independent experiments. GAPDH was the protein loading control. (c) Densitometric analyses of (b) were performed using ImageJ software. The values for control siRNA was normalised to 100 % and the densitometry of the other 3 samples plotted relative to the intensities of GAPDH (protein loading control) in the corresponding lanes. *** *p* ≤ 0.001. (d) Quantitation of ER membrane reorganisation of data shown in Fig. 5d.
